# Outcomes of COVID-19 Infection and Vaccination Among Individuals With Myasthenia Gravis

**DOI:** 10.1001/jamanetworkopen.2023.9834

**Published:** 2023-04-25

**Authors:** Monica Alcantara, Maria Koh, Alison L. Park, Vera Bril, Carolina Barnett

**Affiliations:** 1 Ellen & Martin Prosserman Centre for Neuromuscular Diseases, Toronto General Hospital, University Health Network, University of Toronto, Toronto, Ontario, Canada; 2 ICES, Toronto, Ontario, Canada; 3 Institute of Health Policy, Management and Evaluation, Dalla Lana School of Public Health, University of Toronto, Toronto, Ontario, Canada

## Abstract

**Importance:**

There are limited data regarding COVID-19 outcomes and vaccine uptake and safety among people with myasthenia gravis (MG).

**Objective:**

To investigate COVID-19–related outcomes and vaccine uptake among a population-based sample of adults with MG.

**Design, Setting, and Participants:**

This population-based, matched cohort study in Ontario, Canada, used administrative health data from January 15, 2020, and August 31, 2021. Adults with MG were identified using a validated algorithm. Each patient was matched by age, sex, and geographic area of residence to 5 controls from the general population and from a cohort of individuals with rheumatoid arthritis (RA).

**Exposure:**

Patients with MG and matched controls.

**Main Outcomes and Measures:**

Main outcomes were COVID-19 infection and related hospitalizations, intensive care unit admissions, and 30-day mortality among patients with MG vs controls. Secondary outcomes were uptake of COVID-19 vaccination among patients with MG vs controls.

**Results:**

Among 11 365 233 eligible Ontario residents, 4411 patients with MG (mean [SD] age, 67.7 [15.6] years; 2274 women [51.6%]) were matched to 22 055 general population controls (mean [SD] age, 67.7 [15.6] years; 11 370 women [51.6%]) and 22 055 controls with RA (mean [SD] age, 67.7 [15.6] years; 11 370 women [51.6%]). In the matched cohort, 38 861 of 44 110 individuals (88.1%) were urban residents; in the MG cohort, 3901 (88.4%) were urban residents. Between January 15, 2020, and May 17, 2021, 164 patients with MG (3.7%), 669 general population controls (3.0%), and 668 controls with RA (3.0%) contracted COVID-19. Compared with general population controls and controls with RA, patients with MG had higher rates of COVID-19–associated emergency department visits (36.6% [60 of 164] vs 24.4% [163 of 669] vs 29.9% [200 of 668]), hospital admissions (30.5% [50 of 164] vs 15.1% [101 of 669] vs 20.7% [138 of 668]), and 30-day mortality (14.6% [24 of 164] vs 8.5% [57 of 669] vs 9.9% [66 of 668]). By August 2021, 3540 patients with MG (80.3%) vs 17 913 general population controls (81.2%) had received 2 COVID-19 vaccine doses, and 137 (3.1%) vs 628 (2.8%), respectively had received 1 dose. Of 3461 first vaccine doses for patients with MG, fewer than 6 individuals were hospitalized for MG worsening within 30 days of vaccination. Vaccinated patients with MG had a lower risk than unvaccinated patients with MG of contracting COVID-19 (hazard ratio, 0.43; 95% CI, 0.30-0.60).

**Conclusions and Relevance:**

This study suggests that adults with MG who contracted COVID-19 had a higher risk of hospitalization and death compared with matched controls. Vaccine uptake was high, with negligible risk of severe MG exacerbations after vaccination, as well as evidence of effectiveness. The findings support public health policies prioritizing people with MG for vaccination and new COVID-19 therapeutics.

## Key Points

**Question:**

What are the outcomes of COVID-19 infection and vaccination among individuals with myasthenia gravis compared with the general population and with individuals with another autoimmune disease (rheumatoid arthritis)?

**Findings:**

In this cohort study of 4411 people with myasthenia gravis living in Ontario, Canada, adults with myasthenia gravis who contracted COVID-19 had a higher risk of emergency department visit, hospitalization, and death compared with matched general population controls and controls with rheumatoid arthritis. Patients with myasthenia gravis who were vaccinated had less than half the risk of contracting COVID-19; there was negligible risk of severe myasthenia gravis exacerbations after vaccination.

**Meaning:**

This population-based study suggests that patients with myasthenia gravis had a high risk of serious COVID-19 infection and worse outcomes compared with the general population, supporting the prioritization of this population for vaccination and early therapeutics for COVID-19.

## Teaser

This cohort study investigates COVID-19–related outcomes and vaccine uptake among a population-based sample of adults with myasthenia gravis.

## Introduction

Myasthenia gravis (MG) is an autoimmune disorder caused by antibodies targeting the neuromuscular junction, resulting in fatigue and fluctuating muscle weakness.^1^ The recent COVID-19 pandemic has disproportionally affected people with chronic comorbidities, including MG, who are usually immunosuppressed and, therefore, are at risk of worse infection outcomes.^2^

To our knowledge, there are limited data regarding the risks associated with COVID-19 for people with MG. A cross-sectional study from Italy found no differences in the prevalence of COVID-19 between 162 patients with MG (1.14%; 95% CI, 0.02%-6.17%) and the overall population (0.87%; 95% CI, 0.85%-0.90%) (*P* = .54).^3^ In the CARE-MG (COVID-19 Associated Risks and Effects in Myasthenia Gravis) registry, a physician-reported registry on outcomes of patients with MG and COVID-19, preliminary data from 91 patients showed a high risk of hospitalization (69%), MG exacerbation (40%), and death (24%).^4^ A study of specialized centers in the Czech Republic identified 10 deaths (11%) among 93 patients with MG with COVID-19.^5^ In a matched cohort study from TriNetX COVID-19 Research Network platform, patients with MG had an increased risk of hospitalization (odds ratio, 3; 95% CI, 2.4-3.8) and death (odds ratio, 4.3; 95% CI, 2.9-6.4) compared with the entire cohort of patients with COVID-19 after matching for age and gender.^6^ However, there was no matching for comorbidities, income, or ethnicity, which are factors associated with COVID-19 outcomes. In addition, the study was performed before vaccines were available, so there were no data on vaccine uptake and safety.

We aimed to compare the rates of COVID-19 infections, clinical outcomes, and vaccine uptake of patients with MG with those of a matched control cohort, using population-based data from administrative health databases.

## Methods

This retrospective population-based matched cohort study used provincial administrative health data for all of Ontario, Canada. The data sets and definitions used in this study are in eTable 1 and eTable 2 in Supplement 1. All data sets were linked using unique encoded identifiers and analyzed at ICES, an independent, not-for-profit research institute, whose legal status under Ontario’s health information privacy law allows it to collect and analyze health care and demographic data without consent for health system evaluation and improvement. The use of the data in this project is authorized under section 45 of Ontario’s Personal Health Information Protection Act and does not require review by a research ethics board. This report followed the Strengthening the Reporting of Observational Studies in Epidemiology (STROBE) reporting guideline for observational studies.

### Study Participants and Eligibility Criteria

Patients with MG living in Ontario were identified through a validated algorithm using existing health administrative data at ICES with high sensitivity (81.6%) and specificity (99.9%).^7^ Exclusion criteria were: ineligibility for Ontario Health Insurance Plan (OHIP) coverage within the year before the index date (January 15, 2020), non-Ontario residency, aged younger than 18 years or older than 105 years at the index date, missing geographic data, no contact with the health care system within 5 years prior to index date, and a first MG diagnosis before 18 years of age or during the study period.

Each patient with MG was matched (without replacement) to 5 controls from the general population on age, sex, and geographic area of residence. The general population was identified using the Registered Persons Database, which contains encrypted OHIP numbers and demographic information for all OHIP-eligible individuals in Ontario.

Because our administrative data are limited regarding medications, we also matched to a cohort of individuals with rheumatoid arthritis (RA) to account for a potential association of immunosuppressive treatments with COVID-19 outcomes. The cohort with RA was identified by selecting individuals from the general population cohort who were also in the Ontario RA Database, a validated cohort of Ontarians with RA from April 1988 onward.^8^ The following covariates were compared at baseline: place of residence (rural vs urban), ecological measure of income quintile (Q1-Q5, where Q1 indicates the lowest income quintile and Q5 indicates the highest income quintile), area ethnic diversity quintile (Q1-Q5, where Q1 indicates the lowest diversity quintile and the Q5 indicates highest diversity quintile), chronic comorbidities (asthma, congestive heart failure, chronic obstructive pulmonary disease, hypertension, and diabetes), and Charlson Comorbidity Index score in the previous 5 years.^9,10,11,12,13,14,15^ We also assessed residence in long-term care in the previous 2 years.

### Exposures and Outcomes of Interest

We identified individuals with a positive COVID-19 polymerase chain reaction test result between January 15, 2020, and May 17, 2021, and compared the positivity rate between patients with MG and controls (general population and RA). Among those with a positive test result, we compared rates of emergency department (ED) visits, acute-care hospitalization, and intensive care unit admissions—each occurring between 3 days before and 14 days after the earliest positive COVID-19 test result—as well as death occurring between 7 days before and 30 days after the earliest positive COVID-19 test result. These time windows were used to allow for delays in COVID-19 testing reporting. Emergency department visits, hospitalizations, and intensive care unit admissions were captured up to May 31, 2021, and deaths were identified up to June 16, 2021.

We identified COVID-19 vaccinations among all patients with MG and general population controls from December 2020 (beginning of the vaccination program) until August 31, 2021. To study the safety of COVID-19 vaccination in association with severe MG exacerbations, we assessed hospital admissions with a diagnostic code of MG—indicating MG exacerbation—within 30 days after a dose of COVID-19 vaccine. We also assessed COVID-19 infection rates stratified by vaccination status.

### Statistical Analysis

Mean (SD) values and percentages of baseline characteristics were compared between patients with MG and controls, through standardized differences, with a standardized difference more than 0.10 suggesting an important difference. Prevalence and incidence rates per 1000 person-years and 95% CIs of COVID-19 infection were calculated for patients with MG and matched controls. Among COVID-19–positive individuals, the prevalence and incidence rates of each severe COVID-19 outcome were calculated for patients with MG and matched controls. Incidence rates were compared through hazard ratios (HRs) with MG in the nominator, so an HR greater than 1 indicates higher risk for patients with MG compared with controls; 95% CIs not crossing 1 are considered significant. A Cox proportional hazards regression model was used to estimate an unadjusted HR for COVID-19 infection, comparing patients with MG vs controls, with individuals censored at loss of OHIP eligibility, end of follow-up (May 17, 2021), or 7 days after death to allow for delays in COVID-19 testing or reporting. In addition, among patients with MG, we estimated the HR for COVID-19 infection comparing rural vs urban residence, lowest (Q1-Q2) vs highest (Q3-Q5) income quintiles, and age (≥50 vs <50 years). The percentage of patients and controls who received 1, 2, and 3 COVID-19 vaccine doses by August 31, 2021, was also calculated. All *P* values were from 2-sided tests and, when appropriate, results were deemed statistically significant at *P* < .05. Statistical analysis was performed using SAS statistical software, version 9.4 for UNIX (SAS Institute Inc).

## Results

As of January 15, 2020 (index date), there were 18 051 589 living residents in Ontario. Of these, 6 686 356 (37.0%) were excluded due to lack of OHIP eligibility, non-Ontario residency, and/or being younger than 18 years or older than 105 years at the index date. Among the remaining 11 365 233 individuals, we identified 4411 patients with MG (mean [SD] age, 67.7 [15.6] years; 2274 women [51.6%]), who were matched 1:5 to 22 055 general population controls (mean [SD] age, 67.7 [15.6] years; 11 370 women [51.6%]) and 22 055 controls with RA (mean [SD] age, 67.7 [15.6] years; 11 370 women [51.6%]) (Table 1). In the matched cohort, 38 861 of 44 110 individuals (88.1%) were urban residents; in the MG cohort, 3901 (88.4%) were urban residents. Area income, ethnic diversity quintiles, and residency in long-term care were also well balanced between patients with MG and controls. Patients with MG had higher mean Charlson Comorbidity Index scores and higher prevalence of comorbidities than general population controls; however, these variables were balanced between patients with MG and controls with RA. Table 1 shows baseline demographic characteristic of patients with MG and matched controls.

**Table 1.** Baseline Characteristics of Patients With MG and Matched Controls

Characteristic	Patients with MG (n = 4411)	General population controls (n = 22 055)	Standardized difference^a^	Controls wth RA (n = 22 055)	Standardized difference^a^
Age, mean (SD), y	67.7 (15.6)	67.7 (15.6)	0	67.7 (15.6)	0
Sex, No. (%)					
Female	2274 (51.6)	11 370 (51.6)	0	11 370 (51.6)	0
Male	2137 (48.4)	10 685 (48.4)	0	10 685 (48.4)	0
Urban residence, No. (%)	3901 (88.4)	19 585 (88.8)	0.01	19 276 (87.4)	0.03
Area income quintile, No. (%)					
First (lowest)	938 (21.3)	4371 (19.8)	0.04	4338 (19.7)	0.04
Second	861 (19.5)	4315 (19.6)	0	4475 (20.3)	0.02
Third	901 (20.4)	4419 (20.0)	0.01	4411 (20.0)	0.01
Fourth	808 (18.3)	4292 (19.5)	0.03	4231 (19.2)	0.02
Fifth (highest)	886 (20.1)	4591 (20.8)	0.02	4521 (20.5)	0.01
Area ethnic diversity quintile, No. (%)					
First (lowest)	849 (19.2)	4165 (18.9)	0.01	4403 (20)	0.02
Second	751 (17.0)	3890 (17.6)	0.02	4319 (19.6)	0.07
Third	794 (18.0)	4033 (18.3)	0.01	4111 (18.6)	0.02
Fourth	906 (20.5)	4305 (19.5)	0.03	4189 (19)	0.04
Fifth (highest)	1067 (24.2)	5503 (25.0)	0.02	4804 (21.8)	0.06
Chronic comorbidities, No. (%)					
Asthma	899 (20.4)	2929 (13.3)	0.19	4341 (19.7)	0.02
Congestive heart failure	539 (12.2)	1661 (7.5)	0.16	2369 (10.7)	0.05
Chronic pulmonary obstructive disease	1001 (22.7)	3699 (16.8)	0.15	5170 (23.4)	0.02
Hypertension	2712 (61.5)	12 130 (55.0)	0.13	13 297 (60.3)	0.02
Diabetes	1380 (31.3)	5493 (24.9)	0.14	5949 (27.0)	0.10
Charlson Comorbidity Index score (past 5 y), No. (%)					
Mean (SD), No.	0.8 (1.4)	0.5 (1.1)	0.25	0.7 (1.4)	0.06
0	2976 (67.5)	17 568 (79.7)	0.28	15 366 (69.7)	0.06
1	498 (11.3)	1808 (8.2)	0.10	2866 (13.0)	0.05
2	477 (10.8)	1422 (6.4)	0.16	1805 (8.2)	0.05
≥3	460 (10.4)	1257 (5.7)	0.17	2018 (9.1)	0.09
Residence in long-term care, No. (%)	170 (3.9)	550 (2.5)	0.08	615 (2.8)	0.06

Abbreviations: MG, myasthenia gravis; RA, rheumatoid arthritis.

^a^ Standardized difference of 0.1 or less indicates a good balance of variables.

Between January 15, 2020, and May 17, 2021, 164 patients with MG (3.9%; 25.5 per 1000 person-years), 669 matched general population controls (3.0%; 20.5 per 1000 person-years), and 668 matched controls with RA (3.0%; 23.5 per 1000 person-years) had a positive COVID-19 polymerase chain reaction test result. The unadjusted HR of a positive COVID-19 test result for patients with MG compared with general population was 1.25 (95% CI, 1.06-1.48); compared with matched patients with RA, the HR was 1.24 (95% CI, 1.05-1.48). Among patients with MG, a lower risk of COVID-19 infection was observed for rural vs urban residence (reference group) (HR, 0.49; 95% CI, 0.26-0.92). Aged 50 years or older (HR, 0.81; 95% CI, 0.55-1.19) and low vs high income quintile (HR, 1.02; 95% CI, 0.76-1.39) were not significantly associated with risk of COVID-19 infection among patients with MG.

Among individuals with a positive COVID-19 test result, those with MG had higher rates of severe COVID-19 outcomes than general population controls, including ED visits (36.6% [60 of 164] vs 24.4% [163 of 669]), hospital admissions (30.5% [50 of 164] vs 15.1% [101 of 669]), and death at 30 days (14.6% [24 of 164] vs 8.5% [57 of 669]). When comparing patients with MG with controls with RA, those with MG still had a higher risk of ED visits (36.6% [60 of 164] vs 29.9% [200 of 668]), hospital admission (30.5% [50 of 164] vs 20.7% [138 of 668]), and death at 30 days (14.6% [24 of 164] vs 9.9% [66 of 668]) (Table 2).

**Table 2.** Incidence of Severe COVID-19 Outcomes

Outcome^a^	Patients with MG (n = 164)	General population controls (n = 669)	HR (95% CI)	Controls With RA (n = 668)	HR (95% CI)^b^
ED visit	60 (36.6)	163 (24.4)	1.84 (1.29-2.62)	200 (29.9)	1.49 (1.05-1.48)
Hospitalization	50 (30.5)	101 (15.1)	2.48 (1.61-3.79)	138 (20.7)	1.79 (1.30-2.48)
ICU admission	11 (6.7)	26 (3.9)	2.12 (0.89-5.02)	34 (5.1)	1.63 (0.83-3.23)
Death	24 (14.6)	57 (8.5)	2.11 (1.17-3.78)	66 (9.9)	1.83 (1.17-2.89)

Abbreviations: ED, emergency department; HR, hazard ratio; ICU, intensive care unit; MG, myasthenia gravis; RA, rheumatoid arthritis.

^a^ ED visit, hospitalization, and ICU admission from 3 days before to 14 days after positive COVID-19 test result; death between 7 days before and 30 days after positive COVID-19 test result. Time windows were used to account for delayed test reporting.

^b^ Hazard ratio of incidence rate per 1000 person-years. There was increased risk of ED visits, hospitalizations, and death among patients with MG compared with controls and compared with controls with RA. Total number of individuals per category reflects those with a positive COVID-19 test result during study window.

By August 31, 2021, 3540 patients with MG (80.3%) and 17 913 general population controls (81.2%) had received 2 COVID-19 vaccine doses, 137 patients with MG (3.1%) and 628 general population controls (2.8%) had received 1 dose, and 12 patients with MG (0.3%) and 38 general population controls (0.2%) had received 3 doses. A total of 722 patients with MG (16.4%) and 3476 general population controls (15.8%) remained unvaccinated. Among 3461 first doses among patients with MG, fewer than 6 individuals were hospitalized due to MG within 30 days of vaccination and no patients were admitted after the second dose. Among the 24 patients with MG who died between 7 days before up to 30 days after a positive COVID-19 test result, fewer than 6 (<25%) had received any vaccine dose.

Patients with MG and controls who had received at least 1 dose of COVID-19 vaccine were less likely to contract COVID-19 compared with unvaccinated individuals. This difference was more pronounced among patients with MG (HR, 0.43 [95% CI, 0.30-0.60]) than controls (HR, 0.70 [95% CI, 0.57-0.84]) (Figure).

**Figure.**
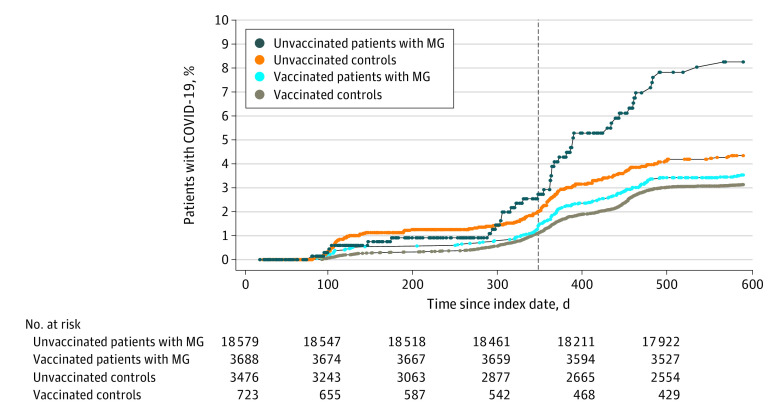
Cumulative Incidence of COVID-19 Infection Among Adults With Myasthenia Gravis (MG) and Controls Stratified According to Vaccination Status Between January 15, 2020, and August 31, 2021 Index date was January 15, 2020. The vertical line shows the start of the vaccination program in Ontario, Canada. The unadjusted hazard ratio for COVID-19 infection was 0.43 (95% CI, 0.30-0.60) among vaccinated vs unvaccinated individuals with MG and 0.70 (95% CI, 0.57-0.84) among vaccinated vs unvaccinated controls.

## Discussion

Our study confirms the higher risk of severe outcomes of COVID-19 infection among people with MG and supports the prioritization of this population for vaccine distribution. In keeping with a US study using administrative data,^6^ we found that patients with MG had a significantly increased risk of hospitalization and death compared with controls. In our study, the absolute 30-day mortality risk for patients with MG was 14.6%, compared with 8.5% for general population controls and 9.9% for controls with RA. Individuals with MG had a higher prevalence of other comorbidities compared with the general population controls, which can also be associated with worse COVID-19 outcomes in this cohort. However, patients with MG were also at higher risk of severe COVID-19 outcomes compared with controls with RA, where comorbidities were well balanced and where immunosuppression is also common, suggesting that there may be factors specific to MG associated with these worse outcomes. The known association of MG worsening with infections, including the risk of MG crisis and respiratory failure, may explain in part the higher risk of admissions and death among patients with MG.^16^

High mortality among patients with MG was also observed in a physician registry–based study with high-quality clinical data.^4^ That study reported a higher death rate compared with our study (24.2% vs 14.6%), which may reflect bias toward reporting more severe cases in comparison with our study, which was population based. In addition, mortality may differ based on epidemiologic considerations and differences in health care systems and access to care, as well as the timing of the study in association with vaccine availability and vaccine uptake.

In our study, most patients who died were unvaccinated, reflecting the study window that covered the first year of the pandemic before vaccines were available; only approximately 6 months of outcomes data with vaccines were available. Therefore, it is likely that the risk of death with broad vaccination programs and the availability of COVID-19 treatments, such as antivirals or monoclonal antibodies, is lower. We found a slightly higher risk of contracting COVID-19 among patients with MG than controls during the study window. This finding is in keeping with previous population-based studies that have shown an increased risk of infections, especially respiratory infections, for people with MG compared with controls.^2^ Immunosuppression likely plays a role in this higher risk; however, in our study, patients with MG also had a higher risk of contracting COVID-19 compared with the controls with RA, another population for whom immunosuppression is common. Therefore, there may be other factors specific to MG accounting for this increased risk. A German cohort study found that, although immunosuppression was an independent risk factor for hospitalization or death due to COVID-19 among people with MG, it was not associated with increased risk of contracting COVID-19, although that study did not have a control group.^17^ As our administrative health data do not have comprehensive treatment information, we cannot assess the role of specific immunosuppressive treatments in the outcomes studied.

We found a high uptake of COVID-19 vaccines; within 9 months of the vaccination program, more than 80% of patients with MG and controls had received at least 2 doses. Even though our study was not designed to assess vaccine efficacy, we did find that unvaccinated patients with MG were more likely to have a positive COVID-19 test result compared with people with MG who had received at least 1 vaccine dose. Although we did not match patients with MG and controls by vaccine status, this finding suggests that vaccination is effective for people with MG, despite immunosuppressive treatments.

In addition, we found that there was a negligible number of MG-related hospital admissions in the 30 days after vaccination, suggesting that the vaccine is safe from an MG perspective. Although we cannot rule out mild MG flare-ups not requiring hospital admission, the risks of severe COVID-19 infection with hospital admission and death were much higher among people with MG in this study. In addition, recent cohort studies have assessed the risk of mild flare-ups of MG after COVID-19 vaccination, which, in general, have been found to be minimal, supporting our findings.^18^ Overall, these findings support guidelines that strongly recommend COVID-19 vaccination for people with MG.

### Limitations and Strengths

Our study has some limitations, including that the follow-up window reflects the characteristics of the initial waves of the pandemic, which were different from the most recent waves and more likely to result in serious respiratory outcomes. In addition, we have scarce data on routine prescriptions—these data are available only for individuals aged 65 years or older or receiving disability benefits—so we could not assess the role of specific immunosuppressants in COVID-19 outcomes. However, using the controls with RA helped to offset this limitation, as most people with RA are immunosuppressed, and the controls with RA were well balanced in comorbidities compared with patients with MG.

Our study also has some strengths, including the use of a validated algorithm to identify patients with MG, as well as the fact that, given the single-payer health care system in Canada, our administrative data cover approximately 95% of people living in the province, regardless of income or location. However, we acknowledge that our algorithm to identify people with MG may have also missed individuals with very mild disease and infrequent contact with the health care system, so our results may not be generalizable to those individuals.

## Conclusions

Our population-based cohort study highlights the increased risk of severe outcomes among people with MG who contract COVID-19, as well as the safety and effectiveness of vaccination for this population. Our results support the prioritization of people with MG for vaccination as well as for consideration of early therapeutics for COVID-19, such as antivirals and/or monoclonal antibodies.

## Notes

Supplement 1. **eTable 1. **Databases Used in the Study **eTable 2. **Databases and Variables Used to Define Study Exposures, Outcomes and Covariates

Supplement 2.
Data Sharing Statement
