# Supplementary material for: Outcomes of COVID-19 Infection and Vaccination Among Individuals With Myasthenia Gravis
Source: JAMA Netw Open. 2023 Apr 25;6(4):e239834. doi: 10.1001/jamanetworkopen.2023.9834 (PMC10130942; doi:10.1001/jamanetworkopen.2023.9834)
Supplement: Supplement 1. — eTable 1. Databases Used in the Study eTable 2. Databases and Variables Used to Define Study Exposures, Outcomes and Covariates [file jamanetwopen-e239834-s001.pdf]

Supplementary Online Content

Alcantara M, Koh M, Park AL, Bril V, Barnett C. Outcomes of COVID-19 infection and vaccination among individuals with myasthenia gravis. *JAMA Netw Open.* 2023;6(4):e239834. doi:10.1001/jamanetworkopen.2023.9834

eTable 1. Databases Used in the Study
eTable 2. Databases and Variables Used to Define Study Exposures, Outcomes and Covariates

This supplementary material has been provided by the authors to give readers additional information about their work.

© 2023 Alcantara M et al. *JAMA Network Open.*

eTable 1. Databases Used in the Study

Database	Time frame	Description
Canadian Institutes of Health Information Discharge Abstract Database (DAD)	1988-2021	Data for acute care institutions in Ontario.
Canadian Institutes of Health Information Same Day Surgery (SDS) database	1991-2021	Data for day surgery institutions in Ontario
National Ambulatory Care Reporting System (NACRS)	2000-2021	Data on emergency department visits, day surgery and outpatient clinic visits
Ontario Health Insurance Plan (OHIP) database	1991-2021	Physicians claims for services provided, including a diagnostic code.
Ontario Drug Benefit Claims (ODB) database	1990-2021	Claims for prescription drugs received under the ODB program. Claims are for persons >= age 65 years, or < 65 years and unemployed, have disabilities, or have high prescription costs relative to net household income.
Registered Persons Database (RPDB)	1991-2021	Demographic information (age, sex, geographic information and vital statistics) about every individual who has ever received a provincial OHIP health card number
Case and Contact Management (CCM)	2020-2021	Data from the central data repository for COVID-19 case and contact management, and reporting in Ontario.
Ontario COVID-19 Vaccine Data (COVAXON)	2020-2021	Information on COVID-19 vaccination events.
COVID19 Integrated Testing Data (C19INTGR)	2020-2021	Created by ICES, this is a comprehensive dataset of all available COVID-19 diagnostic lab results in Ontario, including those from Ontario Laboratories Information System, Distributed testing laboratories, and Public Health CCM
Continuing Care Reporting System (CCRS)	2018-2020	Clinical and demographic information on residents receiving facility based continuing care services.

© 2023 Alcantara M et al. *JAMA Network Open.*

Ontario Asthma Dataset (ASTHMA)	1996-2020	Data on prevalent and incident cases of asthma in Ontario
Ontario Diabetes Database (ODD)	1991-2020	Data on prevalent and incident cases of diabetes in Ontario
Chronic Obstructive Pulmonary Disease (COPD) database	1991-2020	Data on prevalent and incident cases of COPD in Ontario
Ontario Congestive Heart Failure (CHF) database	1991-2020	Data on prevalent and incident cases of CHF in Ontario. The CHF database is limited to those 40 years of age or older.
Ontario Hypertension Database (HYPER)	1991-2020	Data on prevalent and incident cases of hypertension in Ontario
Ontario Rheumatoid Arthritis Database (ORAD)	1993-2020	Data on prevalent and incident cases of rheumatoid arthritis in Ontario
Drugs List (DIN)	1990-2021	The file contains a near exhaustive list of DINs (Drug Identification Numbers) used in Canada from 1990 forward.
Ontario Marginalization Index (ONMARG) (1)	2016	ON-MARG is a geographically (Census) based index developed to quantify the degree of marginalization occurring across the province of Ontario. It is comprised of 4 major dimensions thought to underlie the construct of marginalization: residential instability, material deprivation, dependency and ethnic diversity.
Statistics Canada Census	2016	Used to identify the Census based neighbourhood income quintile

© 2023 Alcantara M et al. *JAMA Network Open.*

Local Health Integration Network (LHIN)	2009	Local Health Integration Networks (LHINs) are not-for-profit corporations that will be responsible for planning, integrating and funding local health services in 14 different geographic areas of the province. LHINs are intended to be the managers for health services that are delivered in hospitals, long-term care facilities, community health centres, community support services and mental health agencies. Used to identify geographic area of residence.

© 2023 Alcantara M et al. *JAMA Network Open.*

eTable 2. Databases and Variables Used to Define Study Exposures, Outcomes and Covariates

**Condition**	**Definition**
Exposure	
Myasthenia gravis (MG) cases	1 hospitalization (DAD) with a MG diagnostic code, or 5 outpatient visits (OHIP) with a MG diagnostic code and 1 relevant service code within 1 year, or 3 prescriptions (ODB), within 1 year.<br><br>DAD<br>ICD-9 diagnostic codes: 358.0, 358.00, 358.01<br>ICD-10-CA diagnostic codes: G70.0, G70.1, G70.2<br><br>OHIP<br>OHIP diagnostic code: 358<br>OHIP service codes: G458, G419<br><br>ODB<br>3 prescriptions for pyridostigmine
Controls	
Rheumatoid arthritis (RA) controls	1 hospitalization (DAD) with an RA diagnostic code, or 3 outpatient visits (OHIP) with an RA diagnostic code within 2 years, with at least 1 diagnosis by a musculoskeletal specialist.<br><br>DAD<br>ICD-9 diagnostic codes: 714<br>ICD-10-CA diagnostic codes: M05, M06<br><br>OHIP<br>OHIP diagnostic code: 714<br>OHIP specialty: Rheumatology, Orthopedic surgery, Internal medicine
Outcomes	
COVID-19 positive PCR test	The date of the earliest occurring positive test result in C19INTGR
COVID-19 emergency department visit	ED visit in NACRS ≤ 3 days before or ≤ 14 days after the earliest positive COVID-19 test

© 2023 Alcantara M et al. *JAMA Network Open.*

COVID-19 acute-care hospitalization	Hospitalization in DAD ≤ 3 days before or ≤ 14 days after the earliest positive COVID-19 test; or in-hospital death ≤ 3 days after hospitalization (DAD) with the earliest positive COVID-19 test ≤ 7 days after death; or Hospitalized=YES in CCM
COVID-19 intensive care unit (ICU) admission	Any hospitalization above with an ICU admission ≤ 3 days before or ≤ 14 days after the earliest positive COVID-19 test
COVID-19 death	Death date in RPDB ≤ 7 days before or ≤ 30 days after the earliest positive COVID-19 test; or Outcome = FATAL in CCM
COVID-19 vaccination status	Date(s) of COVID-19 vaccination in COVaxON
Covariates	
Age at baseline	Age in RPDB
Sex	Sex in RPDB
Geographic area of residence at baseline	Local Health Integration Network of the individual's postal code in RPDB
Long-term care residence within 2 years before baseline	Admission date to LTC in CCRS within 2 years before baseline or a claim in ODB prior to baseline indicating that the claimant is in a LTC facility
Rural residence at baseline	Census
Dissemination area income quintile at baseline	Census
Dissemination area ethnic diversity quintile at baseline	Ethnic Diversity Quintile in ONMARG

© 2023 Alcantara M et al. *JAMA Network Open.*

Hypertension any time before baseline	Hypertension was defined as<br>a) one hospital admission with a hypertension diagnosis, or<br>b) an OHIP claim with a hypertension diagnosis followed within two years by either an OHIP claim or a hospital admission with a hypertension diagnosis.(2)<br><br><u>DAD, SDS</u><br>ICD-9 diagnostic codes: 401, 402, 403 404, 405<br>ICD-10-CA diagnostic codes: I10, I11, I12, I13, I15<br><br><u>OHIP</u><br>OHIP diagnostic codes: 401, 402, 403 404, or 405
Asthma any time before/at baseline	Asthma database was used to identify patients with asthma, based on 2 or more ambulatory care visits and/or 1 or more hospitalizations.(3)<br><br><u>OHIP</u><br>OHIP diagnostic code: 493<br><br><u>DAD</u><br>ICD-9 diagnostic code: 493<br>ICD-10-CA diagnostic codes: J45, J46
Diabetes any time before baseline	ODD was used to identify patients with diabetes, based on 2 OHIP diagnostic codes or 1 OHIP service code or 1 DAD/SDS admission within 2 years.(4)<br><br><u>OHIP</u><br>OHIP diagnostic code: 250<br>OHIP service codes: Q040, K029, K030, K045, K046<br><br><u>DAD, SDS</u><br>ICD-9 diagnostic code: 250<br>ICD-10-CA diagnostic codes: E10, E11, E13, E14

© 2023 Alcantara M et al. *JAMA Network Open.*

Chronic pulmonary obstructive disease (COPD) any time before baseline	COPD database was used to identify patients with COPD, based on 3 or more ambulatory care visits and/or 1 or more hospitalizations within 2 years.(5)<br><br><u>OHIP</u><br>OHIP diagnostic codes: 491, 492, 496<br><br><u>DAD</u><br>ICD-9 diagnostic codes: 491, 492, 496<br>ICD-10-CA diagnostic codes: J41, J42, J43, J44
Congestive heart failure (CHF) any time before baseline	CHF database was used to identify patients with CHF, based on 1 NACRS, DAD, SDS, or OHIP claim and a second claim (from either) in 1 year.(6)<br><br><u>OHIP</u><br>OHIP diagnostic code: 428<br><br><u>NACRS, DAD, SDS</u><br>ICD-9 diagnostic code: 428<br>ICD-10-CA diagnostic codes: I500, I501, I509

© 2023 Alcantara M et al. *JAMA Network Open.*

Charlson comorbidity index score within 5 years before baseline	Charlson Index = Weighted sum of the indicators below identified using DAD and SDS diagnostic codes.(7)<br><br>1. Acute Myocardial Infarction (Weight=1)<br>ICD-9: 410, 412<br>ICD-10-CA: I21, I22, I252<br><br>2. Congestive Heart Failure[a] (Weight=1)<br>ICD-9: 3989, 4020, 4021, 4029, 4040, 4041, 4049, 4254-4259, 428<br>ICD-10-CA: I099, I255, I420, I425-I429, I43, I50, P290<br><br>3. Peripheral Vascular Disease (Weight=1)<br>ICD-9: 0930, 4373, 440, 441, 4431-4439, 4471, 5571, 5579, V434<br>ICD-10-CA: I70, I71, I731, I738, I739, I771, I790, I792, K551, K558, K559, Z958, Z959<br><br>4. Cerebrovascular Disease[a] (Weight=1)<br>ICD-9: 3623, 430-438<br>ICD-10-CA: G45, G46, H340, I60-I69<br><br>5. Dementia (Weight=1)<br>ICD-9: 290, 2941, 3312<br>ICD-10-CA: F00-F03, F051, G30, G311<br><br>6. Chronic Obstructive Pulmonary Disease/other Respiratory[a] (Weight=1)<br>ICD-9: 4168, 4169, 490-505, 5064, 5081, 5088<br>ICD-10-CA: I278, I279, J40-J47, J60-J67, J684, J701, J703<br><br>7. Rheumatic-like Diseases (Weight=1)<br>ICD-9: 4465, 7100-7104, 7140-7142, 7148, 725<br>ICD-10-CA: M05, M06, M315, M32-M34, M351, M353, M360<br><br>8. Ulcers of the Digestive System (Weight=1)<br>ICD-9: 531-534<br>ICD-10-CA: K25-K28<br><br>9. Liver Disease - Mild[ab] (Weight=1)<br>ICD-9: 0702, 0703, 0704, 0705, 0706, 0709, 570, 571, 5733, 5734, 5738, 5739, V427<br>ICD-10-CA: B18, K700-K703, K709, K713-K715, K717, K73, K74, K760, K762-K764, K768, K769, Z944

© 2023 Alcantara M et al. *JAMA Network Open.*

	10. Diabetes - No Chronic Complications[ab] (Weight=1)<br>ICD-9: 2500-2503, 2508, 2509<br>ICD-10-CA: E100, E101, E106, E108, E109, E110, E111, E116, E118, E119, E120, E121, E126, E128, E129, E130, E131, E136, E138, E139, E140, E141, E146, E148, E149<br><br>11. Diabetes with Chronic Complications[ab] (Weight=2)<br>ICD-9: 2504-2507<br>ICD-10-CA: E102-E105, E107, E112-E115, E117, E122-E125, E127, E132-E135, E137, E142-E145, E147<br><br>12. Hemiplegia or Paraplegia (Weight=2)<br>ICD-9: 3441, 342, 343, 3440-3446, 3449<br>ICD-10-CA: G041, G114, G801, G802, G81, G82, G830-G834, G839<br><br>13. Renal (Kidney) Disease[a] (Weight=2)<br>ICD-9: 4030, 4031, 4039, 4040, 4041, 4049, 582, 5830-5837, 585, 586, 5880, V420, V451, V56<br>ICD-10-CA: N032-N037, N052-N057, N18, N19, N250, Z490-Z492, Z940, Z992<br><br>14. Cancer (No secondary found)[b] (Weight=2)<br>ICD-9: 140-172, 174-1958, 200-208, 2386<br>ICD-10-CA: C00-C26, C30-C34, C37-C41, C43, C45-C58, C60-C76, C81-C85, C88, C90-C97<br><br>15. Liver Disease - Moderate or Severe[b] (Weight=3)<br><br>ICD-9: 4560-4562, 5722-5728<br>ICD-10-CA: I850, I859, I864, I982,K704, K711, K721, K729, K765, K766, K767<br><br>16. Cancer (Metastatic - secondary)[b] (Weight=6)<br>ICD-9: 196-199<br>ICD-10-CA: C77-C80<br><br>17. HIV / AIDS[a] (Weight=6)<br>ICD-9: 042-044<br>ICD-10-CA: B20-B22, B24

© 2023 Alcantara M et al. *JAMA Network Open.*

Abbreviations: ICD-9: International Classification of Diseases, 9th Revision; ICD-10-CA: International Classification of Diseases, 10th Revision, Canada

[a] The following hierarchy exists in creating the Charlson indicator variables: “Liver Disease - Moderate or Severe” trumps “Liver Disease – Mild”; “Diabetes with Chronic Complications” trumps “Diabetes - No Chronic Complications”; and “Cancer (Metastatic - secondary)” trumps “Cancer (No secondary found)”.

[b] Note that there are some minor variations from the Quan et al.[7] paper where 5-digit ICD-9 codes were specified (DAD only use ICD-9 codes to the 4th digit) or the ICD-10-CA coding scheme differs from the ICD-10 coding scheme.

© 2023 Alcantara M et al. *JAMA Network Open.*
