# Supplementary material for: Outcomes of COVID-19 Infection and Vaccination Among Individuals With Myasthenia Gravis
Source: JAMA Netw Open. 2023 Apr 25;6(4):e239834. doi: 10.1001/jamanetworkopen.2023.9834 (PMC10130942; doi:10.1001/jamanetworkopen.2023.9834)
Supplement: Supplement 2. — Data Sharing Statement [file jamanetwopen-e239834-s002.pdf]

Data Sharing Statement

Alcantara. Outcomes of COVID-19 Infection and Vaccination Among Individuals With Myasthenia Gravis. *JAMA Netw Open*. Published April 25, 2023. doi:10.1001/jamanetworkopen.2023.9834

Data
Data available: Yes
Data types: Data (not involving human participants), Data dictionary
How to access data: Data dictionary is in supplementary tables.
When available: With publication

Supporting Documents
Document types: None

Additional Information
Who can access the data: The dataset from this study is held securely in coded form at ICES. While legal data sharing agreements between ICES and data providers (e.g., healthcare organizations and government) prohibit ICES from making the dataset publicly available, access may be granted to those who meet pre-specified criteria for confidential access, available at www.ices.on.ca/DAS (email: das@ices.on.ca). The full dataset creation plan and underlying analytic code are available from the authors upon request, understanding that the computer programs may rely upon coding templates or macros that are unique to ICES and are therefore either inaccessible or may require modification.
Types of analyses: for confirmatory analyses
Mechanisms of data availability: access may be granted to those who meet pre-specified criteria for confidential access, available at www.ices.on.ca/DAS (email: das@ices.on.ca).
